# Light Sheet Microscopy for Single Molecule Tracking in Living Tissue

**DOI:** 10.1371/journal.pone.0011639

**Published:** 2010-07-23

**Authors:** Jörg Gerhard Ritter, Roman Veith, Andreas Veenendaal, Jan Peter Siebrasse, Ulrich Kubitscheck

**Affiliations:** Institute of Physical and Theoretical Chemistry, Rheinische Friedrich-Wilhelms Universität, Bonn, Germany; German Cancer Research Center, Germany

## Abstract

Single molecule observation in cells and tissue allows the analysis of physiological processes with molecular detail, but it still represents a major methodological challenge. Here we introduce a microscopic technique that combines light sheet optical sectioning microscopy and ultra sensitive high-speed imaging. By this approach it is possible to observe single fluorescent biomolecules in solution, living cells and even tissue with an unprecedented speed and signal-to-noise ratio deep within the sample. Thereby we could directly observe and track small and large tracer molecules in aqueous solution. Furthermore, we demonstrated the feasibility to visualize the dynamics of single tracer molecules and native messenger ribonucleoprotein particles (mRNPs) in salivary gland cell nuclei of *Chironomus tentans* larvae up to 200 µm within the specimen with an excellent signal quality. Thus single molecule light sheet based fluorescence microscopy allows analyzing molecular diffusion and interactions in complex biological systems.

## Introduction

The observation of single molecules in living cells allows the study of physiological key processes such as signal processing, intracellular transport and gene expression [1]. However, there are no experimental tools to perform such experiments in intact, large biological objects in three dimensions such as tissue or embryos [2]. The internal fluorescence background in such specimen is simply too high making single molecule imaging very problematic if not completely impossible. An elegant solution presents light sheet based fluorescence microscopy (LSFM), which is an adaptation of classical “Ultramicroscopy” [3] to fluorescence microscopy. Although LSFM is a new technique, it has intensely been developed from its invention until now and had already a remarkable success in imaging complex three dimensional (3D) multicelluar organisms, e.g. embryos, zebrafish or mouse embryos, *in vitro* and *in vivo* [4]–[14].

LSFM uses a thin, focused light sheet to illuminate the sample orthogonally to the detection pathway. Therefore only a narrow region near the object plane of the observation objective lens is illuminated and fluorophores outside this plane are not excited and do not generate a background signal. Furthermore, fluorophores outside the focal plane are not photo-damaged as they would be upon epi-illumination. These instrumental characteristics produce an optical sectioning effect, improved contrast and allow very long observation times of sensitive samples [15].

However, LSFM is not limited to the optical sectioning of comparatively large samples. Here we demonstrate that single molecule imaging in solution and especially in living tissue substantially profits from using light sheet illumination, because single molecule experiments are extremely sensitive in terms of background fluorescence and photobleaching. Other optical sectioning schemes like total-internal-reflection microscopy (TIRF) [16] or highly inclined laminated optical sheet microscopy (HILO) [17], [18] already became very successful in this field, but because of their limited penetration depth they are not suitable for single molecule tracking in extended 3D samples. Here, light sheet based microscopy would be an ideal solution for single molecule experiments in thick specimen [19], [20]. We describe the development and application of a light sheet based fluorescence microscope, which combines the benefits of optical sectioning, the detection efficiency of high numerical aperture (NA) objective lenses (NA>1.0) and parallel image acquisition. Thus it became possible to image the dynamics of single molecules with unprecedented speed and precision *in vitro* and *in vivo* deep within a living specimen. The utility of this instrument was first shown by imaging and tracking of different molecular species in buffer at the single molecule level and the imaging contrast was directly compared to data acquired with classical epi-illumination. The advantages for *in vivo* single molecule imaging in 3D-extended biological tissue were demonstrated by observing tracer molecules within salivary gland cell nuclei of *C. tentans* larvae. From this experiment we could determine the effective intranuclear viscosity, a parameter which affects the mobility of all molecules and particles in the nucleus. Then we studied the trafficking of single, native mRNA molecules in *C. tentans* salivary gland cell nuclei, which were made visible by a fluorescently labeled RNA binding protein, hrp36. This protein is the *C. tentans* homologue of the mammalian RNA binding protein hnRNP A1 [21], [22]. Our new imaging approach yielded images of single RNA particles with a superb contrast, which allowed their tracing at high frame rates and at high spatial resolution about 100 to 200 µm deep within the salivary gland.

## Results

### Instrument design

**Figure 1:**
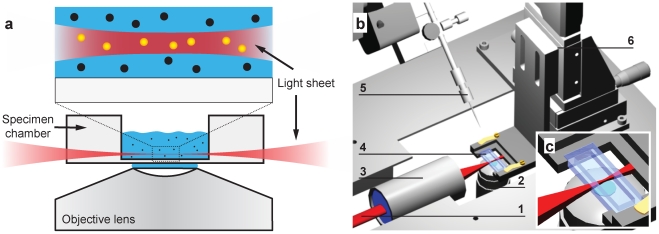
Concept and experimental realization of single-molecule light sheet based fluorescence microscopy. (**a**) The illuminating laser light sheet was focused into a glass sample chamber, whose bottom had standard cover slip thickness. Single molecule fluorescence was collected by a high NA water immersion objective and detected by a fast EMCCD. Due to the optical sectioning effect only fluorescence in the focal region was excited and no out-of-focus fluorescence contributed to the image (enlarged view). (**b**) Experimental realization on the stage of an inverse microscope. An elliptical laser beam (1) is focused by the illumination objective (3) into the sample chamber (4) creating the light sheet. Imaging occurred perpendicular to the illumination axis by a high NA lens (2). Specimen could be moved through the light sheet by a motorized translation stage (6). Optionally, specimen could be microinjected (5). (**c**) Enlarged view of the specimen chamber and the light sheet.

Our instrument is quite different from other LSFMs, because several new design challenges had to be met. While most existing setups [9]–[11], [20], [23] use large specimen chambers together with water-dipping objectives mounted horizontally to image 3D extended specimen, we chose to exploit the stage of a commercial inverted microscope. Furthermore, we used a high NA water immersion objective lens for imaging, which was needed to maximize the light detection efficiency (**Fig. 1**). The custom-built sample chamber featured a glass bottom with standard coverslip thickness (0.17 mm), had glass walls such that the illumination light could be introduced from the side and was placed on a motorized stage (**Materials and Methods** **S1**). The light sheet had to be created within the working distance of 280 µm of the detection objective and was produced by a dedicated illumination objective lens with a high NA in order to achieve a thin light sheet with an axial extension of only 3 µm full-width-at-half-maximum (FWHM). The high NA of the illumination objective resulted in a strong convergence of the focused beam and a corresponding short working distance of the illumination objective. A special long working distance illumination objective (10×; NA = 0.28; working distance, 33.5 mm; Mitutoyo, Japan) met all construction conditions. We built the light sheet illumination on a standard microscope body in order to increase its usability, because this allowed us to switch between fluorescence imaging exploiting the light sheet advantages and standard microscopy techniques like phase or differential interference contrast (DIC). We chose an inverted stage because the specimen chamber had to be accessible for a microinjection device (**Fig. 1b**).

### Light sheet characterization

**Figure 2:**
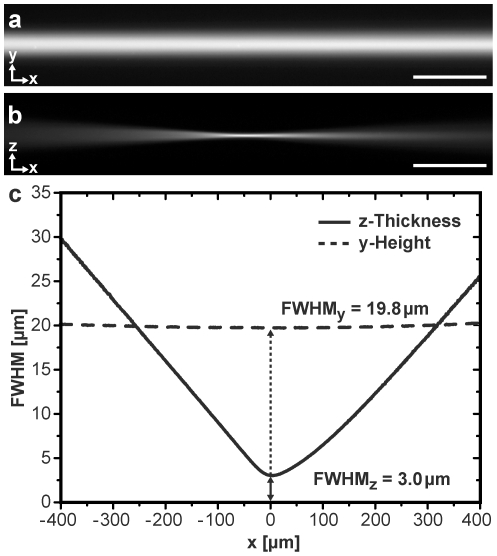
Dimensions of the light sheet. The red light sheet (λ = 638 nm) was formed in a solution with a 100 µM concentration of ATTO647N. It was imaged with a 10× NA 0.3 objective by a slow-scan CCD-camera (**Materials and Methods** **S1**). Lateral (**a**) and axial (**b**) extension of the light sheet. Scale bars, 100 µm. (**c**) Full-width-at-half-maximum (FWHM) values of the light sheet along the illumination (x-) axis. Light sheet geometries of all excitation wavelengths were summarized in **Table 1**.

The actual light sheet dimensions defined the field of view and optical sectioning capability of the microscope. To measure the light sheet dimensions a aqueous solution of ATTO647N was filled into the sample chamber at a concentration of 100 µM. Its illumination directly revealed the 3D extensions of the light sheet, which were imaged and quantified (**Fig. 2**). The minimal FWHM along the illumination axis was 19.7±0.1 µm. Upon turning the elliptical illumination beam by 90° the thickness of the light sheet could directly be visualized and analyzed in a similar manner. Its axial width was 3.0±0.1 µm FWHM for an excitation wavelength of 638 nm. The extension of the usable light sheet along the illumination axis can be defined by twice the Rayleigh length of the Gaussian beam, so that the field of view for optimal contrast was approximately 84 µm×20 µm. Similarly, for excitation wavelengths of 488 nm and 532 nm the axial FWHM values were determined as 2.9±0.1 µm and 3.0±0.1 µm, respectively (**Table 1**). The optical sectioning thickness was comparable for all three wavelengths as it was expected for an achromatic illumination.

**Table 1:** Light sheet dimensions for different excitation wavelengths.

λ [nm]	Axial width (z) [µm]	Y-height [µm]
637	3.0±0.1	19.7±0.1
532	3.0±0.1	19.6±0.1
488	2.9±0.1	19.6±0.1

### Imaging of single molecules in solution

To demonstrate the sensitivity and speed of the instrument we investigated the mobility of single molecules in aqueous solution. An increased contrast was expected due to the lack of out-of-focus fluorescence, such that it should be possible to image and track smaller (and faster) molecules at the single molecule level than ever before [24].

**Figure 3:**
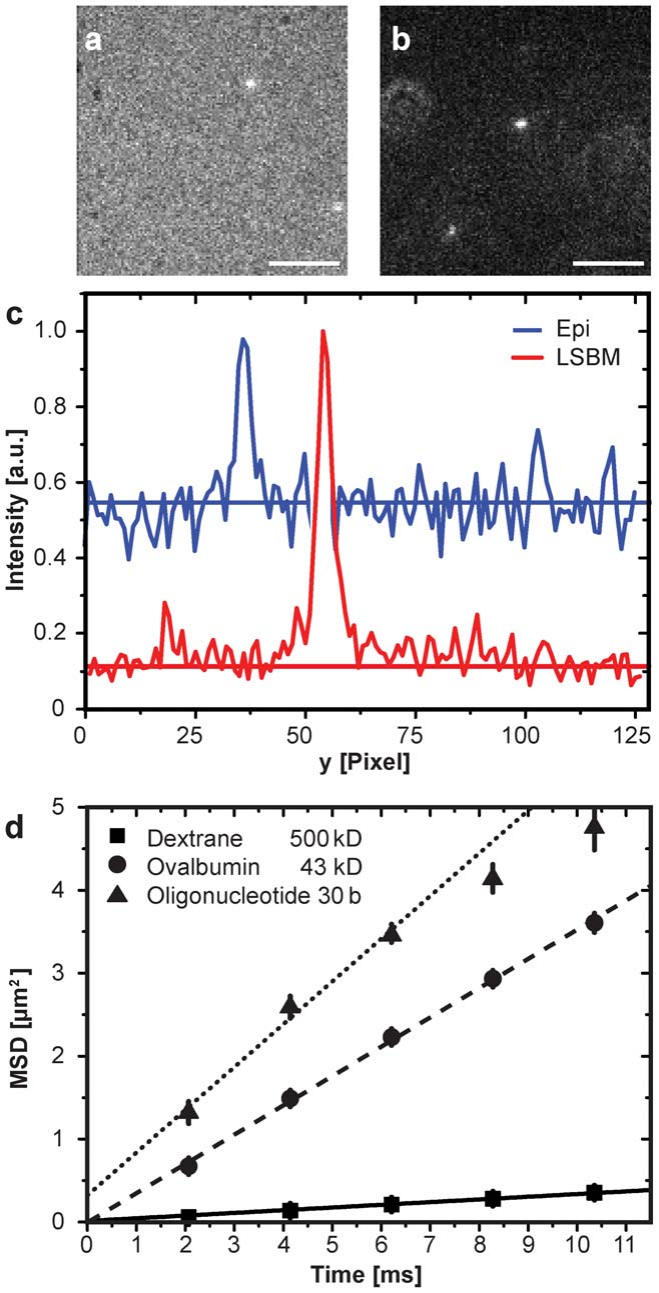
Single molecule visualization and tracking in solution. (**a**) Standard epi-illumination fluorescence image of single ATTO647-labelled dextran molecules (MW 500 kDa) diffusing in transport buffer acquired at an exposure time of 10 ms. Scale bar, 5 µm (**b**) The same sample was imaged upon light sheet illumination demonstrating the striking contrast improvement (see **Movie S1**). Scale bar, 5 µm. (**c**) Intensity profile along a vertical line through the central pixels of the two brightest molecules shown in (a) and (b) illustrating the contrast improvement. (**d**) MSDs as a function of time for different molecules; dextran 500 kDa (boxes), ovalbumin (circles), and a 30mer oligonucleotide (triangles). Movies were recorded for all three species with 483 Hz. The data were fitted by lines to determine the diffusion coefficients. The error bars represent the standard deviation. The results were summarized in **Table 2**. (see **Discussion** **S1**).

For a quantitative comparison between LSFM and standard epi-illumination we compared the image contrast in image sequences of single, fluorescently labeled 500 kDa dextran molecules recorded at almost 100 Hz (**Fig. 3** and **Movie S1**). As expected the contrast was clearly superior (0.97) when the light sheet illumination was used versus epi-illumination (0.37) for the given tracer concentration (**Materials and Methods** **S1**). The signal-to-noise-ratio (SNR) increased by a factor of 4 yielding a significantly improved localization precision [25].

We imaged and analyzed three different molecular species with different sizes and shapes, namely 500 kDa dextran molecules with a Stokes radius of 26.5 nm [21], ovalbumin (MW 43 kDa) with a Stokes radius of 2.4 nm [24], and fluorescently labeled 30mer oligonucleotides (long-rod, with equatorial radii a = 5 nm and b = 0.5 nm [26]). The theoretically expected diffusion coefficients covered three orders of magnitude (**Table 2**). Respective image sequences were recorded at 483 Hz; in all cases we could observe and follow the trajectories of single probe molecules (**Movie S2**). The mean-square-displacements (MSDs), 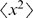, were extracted from the single molecule trajectories and plotted as a function of time, t, to determine the diffusion constants, D, of the probe molecules according to 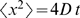 (**Fig. 3**). In this manner we found diffusion coefficients for 500 kDa dextranes, ovalbumin and 30mer oligonucleotides of D_dextran_ = 8.2±0.1 µm^2^/s, D_ova_ = 88±2 µm^2^/s and D_30mer_ = 130±14 µm^2^/s. To validate these data we performed measurements by fluorescence correlation spectroscopy (FCS) (**Table 2**). In all cases we found an excellent correspondence between single molecule tracking, FCS and the theoretically estimated diffusion coefficients. These values measured at a viscosity of 1cP also served as references for the measurement of the effective viscosity within the nucleoplasm of salivary gland cell nuclei of *C. tentans* larvae.

**Table 2:** Diffusion constants of various probe molecules measured by single-molecule LSFM in aqueous solution.

Molecules	Radius [nm]	D_MSD_ [µm^2^/s]	D_FCS_ [µm^2^/s]	D_Theory_ [µm^2^/s]
500 kDa dextran	26.5	8.2±0.1	7.5±0.8	8.1
Ovalbumin	2.4	88±2	92±5	89
Oligonucleotide	a = 5, b = 0.5	130±14	120±2	120

**Table 3:** Mobility of 500 kDa dextran molecules and mRNPs inside living salivary gland cell nuclei.

	500 kDa dextran	mRNP particle
D_1_ [µm^2^/s]	2.3±0.2	2.0±0.2
Fraction 1	64±3%	45±3%
D_2_ [µm^2^/s]	0.5±0.1	0.50±0.05
Fraction 2	34±3%	45±2%
D_3_ [µm^2^/s]	-	0.08±0.01
Fraction 3	-	10±1%

### *In vivo* tracking of single mRNP particles in the nucleus

**Figure 4:**
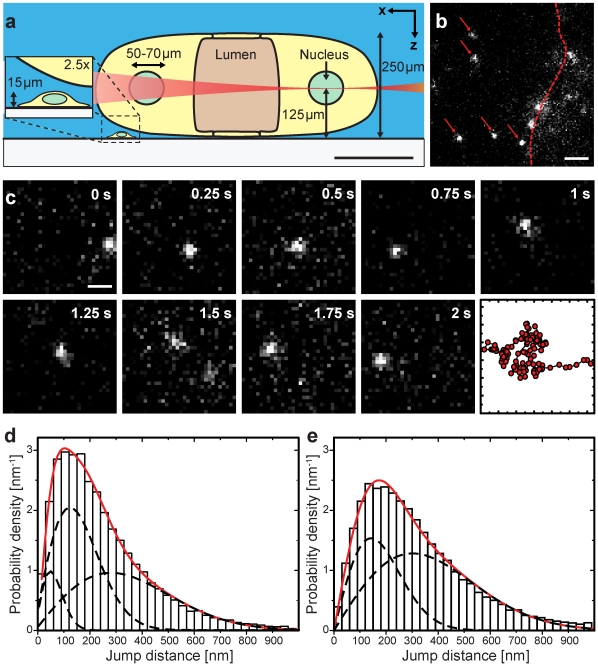
Single molecule tracking in the living salivary gland cell nucleus of the *C.tentans* larvae. (**a**) Scheme of the light sheet illumination of a salivary gland cell nucleus. The salivary glands are a living tissue with dimensions much larger than that of e.g. cell culture cells. Scale bar, 200 µm. (**b**) Typical image showing single diffusing BR mRNPs (red arrows) labelled with microinjected hrp36. The dashed line indicates the position of the nuclear envelope. Scale bar, 3 µm. (**c**) Time series of a moving mRNP particle marked by single ATTO647N-labelled hrp36 molecules inside the nucleus. The last panel shows the complete trajectory. Frame rate, 49.46 Hz; Scale bar 1 µm. (**d**) Normalized jump distance distribution of BR mRNP particles and microinjected 500 kDa dextran molecules (**e**) inside salivary gland cell nuclei for a time interval of 20 ms. The red line showed the complete fitting function and the dashed lines indicate the contributions of the single components (see **Materials and Methods** **S1**). Fitting results were summarized in **Table 3**.

In a next step we employed LSFM for *in vivo* imaging within a 3D extended biological specimen with large dimensions compared to plain monolayer culture cells. As a suitable model system we chose the salivary gland cell nuclei of larvae of the dipteran *C. tentans* [27]–[29]. This system was well suited to demonstrate the imaging capabilities of the single molecule LSFM, because the salivary glands are an intact, large and living tissue. The salivary glands have a complex structure and dimensions of roughly 700 µm×2000 µm×250 µm as sketched in **Fig. 4a**. The gland cells contain large nuclei with diameters of 50–70 µm (**Fig. 4a**). Each gland cell nucleus contains four polytene chromosomes being roughly 10 µm in diameter. Each polytene chromosome is made up of 8000 to 16000 perfectly aligned chromatids [29], which form a distinct chromosome band structure. The remaining nucleoplasm is devoid of chromatin [30]. This is an ideal system to study the regulation of mRNA trafficking without the possibly retarding effect of chromatin (see **Figure S1**).

Chromosome IV contains two especially active transcription sites, the so called Balbiani Rings (BRs) 1 and 2. Three genes with a length of up to 40 kb are transcribed here and are processed to large BR mRNA particles (BR mRNPs) with a diameter of roughly 50 nm, which move randomly in the nucleoplasm after their release from the gene. In the nucleoplasm of the salivary gland cell nuclei the BR mRNPs have a density of 10–100 particles per µm^3^ and form about 90% of the present mRNPs [31]. The glands were dissected out of the larvae and placed inside the specimen chamber for microscopy. In this large biological system visualization of single, fluorescently labeled BR mRNPs and tracking their movements was problematic, since the nuclei had a distance to the cover slip of 100 to 200 µm. In a conventional fluorescence microscope single molecule imaging at such a depth within the specimen is almost impossible, because the SNR is low due to a high fluorescence background (see **Figure S2**).

For labeling of the mRNPs we used the characteristics of the RNA-binding protein hrp36, which is an important structural component of BR mRNPs with about 120 copies contained in a single BR mRNP [32]. Hrp36 is part of the mRNPs from the genes in the nucleus to the polysomes in the cytoplasm and it is associated with the mRNPs even during passage of the particles through the nuclear pore complex. Within living salivary glands BR mRNPs are continuously formed in the nuclei, and therefore we could label the BR mRNPs by *in vivo* microinjection of fluorescent ATTO647N-labeled hrp36 (**Materials and Methods** **S1**). The formation of BR mRNPs takes approximately 10 minutes. Localization and microinjection of the nuclei inside the salivary gland cells was performed using bright-field imaging, since the nuclei were easily distinguishable from the cellular interior.

Immediately after microinjection single hrp36 could not be imaged at a frame rate of 50 Hz, because this frame rate was not high enough to image single, small proteins. However, after about 10 min single hrp36 signals became visible. Presumably, they were then incorporated into the 50 nm sized BR mRNPs, which diffused at a much lower rate [21]. Hence, in this manner the movements of single, native BR mRNPs in the nucleoplasm could be studied. We compared the integrated signal intensity of BR mRNPs with that of single ATTO647N molecules, and concluded that the mRNPs carried single Atto647N-labeled hrp36 molecules. Due to its optical sectioning capability LSFM was especially well suited for this task. Only BR mRNPs in the focal region were illuminated and visualized (Fig. 4b). This resulted in excellent SNRs and clearly longer trajectories of the BR mRNPs (**Fig. 4c** and **Movie S3**) than in previous experiments [21]. In order to analyze the mRNP mobility, we determined the positions along the single particle trajectories with a localization precision of σ = 40±12 nm (**Materials and Methods** **S1**), and plotted the jump distances between consecutive frames in histograms (Fig. 4d). These histograms can be analyzed by a multi-component analysis. We found three different mobility fractions of the BR mRNPs. A fast fraction (45%) with a diffusion coefficient of D_1_ = 2.0±0.2 µm^2^/s, a slower fraction (45%) with a D_2_ = 0.5±0.05 µm^2^/s and a strongly retarded fraction (10%) with a D_3_ = 0.08±0.01 µm^2^/s.

To compare the mRNP mobility with inert molecules of a similar geometrical size we microinjected fluorescent dextran molecules (MW 500 kDa) with a Stokes radius of 26.5 nm into salivary gland cell nuclei. Using LSFM it was straightforward to image these dextran molecules within the nuclei with an excellent signal quality, and to track them for extended time periods (**Movie S4**). From the resulting trajectories we determined the jump distance histogram as described above. In contrast to the situation in buffer the analysis now revealed the existence of two different mobility components, a fast one (64%) with D_fast_ = 2.3±0.2 µm^2^/s and a slower one (36%) with D_slow_ = 0.5±0.1 µm^2^/s (**Fig. 4e**). A comparison of D_fast_ with the diffusion coefficient measured in buffer solution yielded the effective nuclear viscosity in these cell nuclei as *η* = 3.6±1 cP corroborating previous FRAP and single particle tracking results [21]. We speculated that the occurrence of the second, reduced diffusion constant D_slow_ was due to the molecularly crowded intranuclear environment [33]. The comparison of dextran and mRNP mobility suggested that D_1_ and D_2_ were related to the diffusion of the mRNPs in the crowded nuclear interior, because they were almost identical to D_fast_ and D_slow_ of the tracer. However, the third, quite slow mRNP diffusion coefficent, D_3_, was not observable for the inert dextran. We concluded that this mobility component was caused by interactions of the BR mRNPs with large intranuclear structures.

## Discussion

Single molecule observation in large, living samples is problematic. Single molecule microscopy using conventional epi-illumination suffers from out-of-focus fluorescence, which reduces the SNR and decreases the localization precision. Illumination schemes like TIRF or HILO [16]–[18] are highly selective and yield good SNRs, but they are restricted to excitation volumes close to the glass-sample interface. Single-molecule LSFM allows optical sectioning deep inside biological tissue and the use of high NA water-immersion objective lenses, both beneficial for optimal signals. Biological, fluorescent molecules as small as a few nanometers were accessible to single molecule observation in aqueous solution, and we succeeded to track molecules with a diffusion coefficient of greater than 100 µm^2^/s. The LSFM is therefore an excellent tool to perform single molecule studies in living tissue. Moreover, the method has great potential for super resolution imaging and 3D tracking of single molecules in large, living specimens such as embryos at a millisecond time scale.

## Materials and Methods

### Optical setup

We used an Axiovert 200 (Carl Zeiss MicroImaging GmbH, Göttingen, Germany) and replaced the microscope stage with a custom-built stage, which allowed illumination perpendicular to the detection axis. The lateral position of the specimen chamber was adjusted by micrometer screws (BM11.16, Newport, Darmstadt, Germany), and axially by a motorized stage (M105.1B translation stage with DC-Mike linear actuator M232-17 from PI, Karlsruhe, Germany). The specimen chamber was made of BK7 glass and was especially manufactured for our purposes (Hellma, Müllheim, Germany). The internal dimensions of the chamber were 4 mm×20 mm×2 mm. The wall thickness was 2.5 mm and the bottom had standard cover slip thickness, 0.17 mm.

For fluorescence excitation three laser lines were used: A 488 nm DPSS laser (Sapphire-100, Coherent, Germany), a 532 nm solid state laser (LaNova50 Green, Lasos, Germany) and a 638 nm laser diode module (Cube635-25C, Coherent, Germany). All three laser lines were guided with dichroic beam splitters to an acousto-optical tunable filter (AOTF.nC 1001, Opto-Electronics, France). The AOTF selected laser lines, and defined illumination durations and intensity. After the AOTF the light was coupled into a mono-mode fiber (kineFlex, Point Source, Hamble, UK) and guided onto the optical table. Here the elliptical Gaussian illumination beam was formed by a cylindrical Galilean beam expander. It consisted of a convex cylindrical lens with a focal length of f = 250 mm and a concave lens with a focal length of f = −38.1 mm (CKX540-C and CKV522-C, Newport, Darmstadt, Germany). As illumination objective we used a plan apochromat 10×, NA 0.28 long working distance objective lens (Mitutoyo, Japan).

Fluorescence signals were collected with a 40×, NA 1.2 water immersion objective lens (C-Apochromat, Carl Zeiss MicroImaging GmbH, Göttingen, Germany), or a 10×, NA 0.3 objective lens (EC Plan-Neofluar, Zeiss). In the emission beam path respective narrow bandwidth notch filters were employed (Semrock, Rochester, USA). For imaging an EMCCD camera with 128×128 pixels was used (iXon BI DV-860, pixel size 24 µm, Andor Technologies, Belfast, Ireland). A 4× magnifier (Carl Zeiss MicroImaging GmbH, Göttingen, Germany) was added in front of the camera, which resulted in an objective field pixel size of 150 nm for the 40×, NA 1.2 objective lens. The dimensions of the light sheet were measured with a slow-scan CCD camera (Axiocam MRm, Carl Zeiss MicroImaging GmbH, Göttingen, Germany) with a pixel size of 645 nm for the 10×, NA 0.3 objective lens.

### Determination of light sheet thickness

ATTO647N (ATTO-TEC GmbH, Siegen, Germany) was diluted in buffer at a concentration of 100 µM and filled into the specimen chamber. Upon laser excitation a homogenous image of the light sheet was created (**Fig. 2**). In standard configuration the extension in the y-direction of the light sheet was imaged. Rotating the cylindrical lenses by 90° rotated the elliptical illumination beam, and therefore the z-width of the light sheet could be imaged. Images were taken for all excitation laser lines. Excitation of the ATTO647N by 488 and 532 nm occurred due to a small absorption band in the blue-green range of the dye spectrum. The light sheet was imaged by a 10× NA 0.3 objective (EC Plan-Neofluar, Zeiss) and detected by an Axiocam MRm (Carl Zeiss MicroImaging GmbH, Göttingen, Germany). For both y- and z-directions the FWHM of every vertical pixel line in the image was plotted versus the position on the illumination axis, which allowed the determination of the light sheet waists (**Fig. 2**).

### Single molecule imaging and analysis

For imaging of single molecules in solution the molecules were diluted to a final concentration of 100 pM in transport buffer. Fluorescence signals were detected with a 40×, NA 1.2 water immersion objective lens at room temperature. All movies were recorded at a frame rate of 483.09 Hz corresponding to an exposure time of 2 ms per frame. Fluorescence was excited by the 638 nm laser line.

Before tracking a background subtraction and 3×3 Gaussian filtering was performed on the image stacks. Single molecule signals were identified by the threshold algorithm of the tracking software Diatrack v3.03 (Semasopht), which was also used for trajectory definition. To exclude occasionally occurring aggregates of ovalbumin or oligonucleotides from the analysis unusually bright signals and unusually slow moving particles were excluded from the analysis.

From the resulting trajectories the mean-square-displacement (MSD) was calculated and plotted versus time t. The data were fitted according to <x^2^> = 4Dt to determine the diffusion coefficient D. This was done with Origin 8.0 PRO (OriginLab Corporation, Northampton, USA).

To calculate the theoretical diffusion coefficient we used the Stokes-Einstein equation [34].
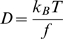
(1)Here *D* is the diffusion coefficient, *k_B_* the Boltzmann constant, *T* the temperature and *f* the frictional drag coefficient. For a sphere with radius r such as the 500 kDa dextran and ovalbumin, *f* is defined as [34]:
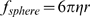
(2)For a randomly moving ellipsoid, such as the 30b oligonucleotide, it is defined as:
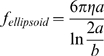
(3)
*η* is the viscosity of the medium, a and b the semi-major and -minor axes of a prolate ellipsoid of revolution. The according radii were obtained from the literature (500 kDa dextran [21], [35] and oligonucleotide [26]) or calculated according to the equation [24] (ovalbumin):
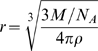
(4)Here *M* is the molar mass, *N_A_* the Avogadro constant and *ρ* refers to the protein density (1.2 g cm^−3^) [36].

The contrast *C* of image sequences of 500 kDa dextran molecules was determined according to this definition.
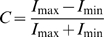
(5)
*I_max_* designates the intensity of a molecule in focus above background *I_min_*. *I_max_* was determined by fitting a 2D-Gaussian to the signals. *I_min_* was determined by measuring the mean intensity in areas where no molecules were seen. All image sequences were recorded with an exposure time of 10 ms, which equates to frame rate of 98.91 Hz.

### *In vivo* imaging and analysis

Imaging of the 500kDa dextran molecules and the labeled mRNP particles in salivary gland cell nuclei of *C. tentans* larvae were performed with a 40×, NA 1.2 water immersion objective lens at room temperature. Image integration time was 20 ms corresponding to a frame rate of 49.46 Hz. Fluorescence was excited by the 638 nm laser line.

All image stacks were analyzed with the tracking software Diatrack v3.03 (Semasopht). Background subtraction and Gaussian filtering was performed before signal identification. To localize the particle, every particle signal was fitted by a 2D Gaussian. Sequences of single, localized events were combined to trajectories.

The determination of the 2D-localization precision of moving BR mRNPs in the salivary gland cells was achieved using the equation[37],
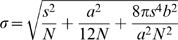
(6)where *s* is the standard deviation of the point-spread function, *a* the pixel size, *b* the standard deviation of the background and *N* the total number of photons contributing to a signal. To determine the latter value the average number of the integrated intensity values for a signal was determined. Finally the total number of photons per signal was calculated considering the count-to-photoelectron conversion factor for the used iXon camera [38].

Out of the trajectories the jump distance from one frame to the next frame was calculated for every trajectory. These jump distances were plotted in a normalized histogram (**Fig. 4d & e**) and fitted according to the following equation [39]:
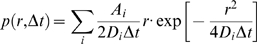
(7)This equation represents the theoretical jump distance probability density function for *i* diffusive species. A*_i_* is the fractional amount of a single species and D*_i_* the respective diffusion coefficient. *r* is the jump distance covered in the time interval Δt. In a complex medium like a cell nucleus a single, distinct diffusion coefficient was not expected and the equation above allowed a multi-component analysis of the *in vivo* data. The calculations and data fitting were performed by Origin 8.0 PRO (OriginLab Corporation).

We measured a reference diffusion coefficient in aqueous solution (1 cP) and also the diffusion coefficient in the nucleoplasm. For the calculation of the viscosity of the nucleoplasm we used **Eq. 1**.

### Microinjection of *C.tentans* salivary glands

*Chironomus tentans* were raised as described [21]. Salivary glands were isolated from fourth instar larvae and microinjected with an Eppendorf injection and micromanipulation setup using a holding pressure of 25 hPa and manual injection procedure.

### Buffer and reagents

Phosphate buffered saline (PBS) was prepared from a commercially available stock solution (Biochrom AG, Berlin, Germany). Transport buffer contained 20 mM HEPES/KOH, pH 7.3, 110 mM potassium acetate, 5 mM sodium acetate, 2 mM magnesium acetate, 1 mM EGTA, and 2 mM DTT. Amino-derivatized dextran (molecular mass 500 kDa; Invitrogen, Germany) was dissolved in 0.1 M NaHCO_3_, pH 8, and fluorescence labeled with a 5-fold excess of ATTO647 succidinimidyl ester (ATTO-TEC GmbH, Siegen, Germany). Chicken egg Ovalbumin (molecular mass ∼43 kDa; Sigma-Aldrich, Germany) was solved in PBS containing 1mM TCEP (tris(2-carboxyethyl)phosphine; Sigma-Aldrich, Germany) and labeled with ATTO647N maleimide (ATTO-TEC GmbH, Siegen, Germany). Preparation of ATTO647N-labeled hrp36 was according to [21]. All labeling reactions were set up at room temperature for 2 hours and free dye was removed by gel filtration on a BioRad-P6 desalting column (MW cut off 6 kDa; BioRad, Munich, Germany). Labeled probes were finally size-fractionated on a Superose 12 column to remove aggregates and smaller fragments. The 2′-O-methyl RNA oligonucleotide homologue to the repetitive portion of the BR 2.1 mRNA was obtained from IBA BioTAGnologies (Göttingen, Germany). It comprised 30 bases (ACT TGG CTT GCT GTG TTT GCT TGG TTT GCT) and contained a 5′ fluorescence label (ATTO647N). To check for purity the oligo was resolved on a 15% polyacrylamide gel prior to use.

### Fluorescence correlation spectroscopy

FCS measurements were performed using a Zeiss Confocor I microscope setup. For calibration of the beam width ATTO647N-maleimide dye molecules (MW 870 Da; ATTO-TEC GmbH, Siegen, Germany) in buffer were used. The diffusion coefficient of ATTO655-maleimide dye (MW 810 Da) has been measured previously with high precision [40], and a diffusion coefficient of 400 µm^2^/s was determined. Since both dyes were comparable in molecular weight, we assumed a diffusion coefficient of 400 µm^2^/s for ATTO647N-maleimide as well. This assumption was corroborated by the characteristic diffusion times for the two dyes of 63 and 62 µs, respectively. Calibration of the beam width was done prior to the measurements. Before a single FCS run, a z-scan was recorded to ensure that the measurement was performed in the solution not close to the coverslip. Data analysis was performed using FCS ACCESS (Evotec, Hamburg, Germany). The theoretical autocorrelation curve for 3D diffusion of up to 3 mobility components is,
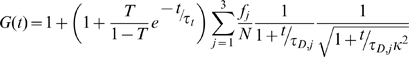
(8)where *f_j_* are the fractions corresponding to the different diffusion times *τ_D.j_*, *N* is the total number of fluorescent molecules, *T* is the ratio of triplet state, *τ_t_* is the triplet time and *κ* is the axial e^−2^ beam radius of the laser beam divided by the lateral e^−2^ beam radius. *κ* was usually close to 5. *τ_D_* is related to the diffusion coefficient by
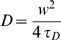
(9)where *w* is the lateral e^−2^ beam radius of the laser spot. Laser illumination was performed with 633 nm light.

ATTO647-labeled 500 kDa dextran molecules were diluted 1∶1000 from the stock in PBS, followed by centrifugation at 22000×g for 45 minutes. Before measuring, a MatTek dish (MatTek Corp., Ashland, USA) was coated with bovine serum albumine (10g/L). After removing the coating solution, 300 µL dextran solution was added and covered with a coverslip to prevent evaporation. Single FCS runs with 10 to 30 seconds were performed and average values were calculated. ATTO647N-labeled ovalbumin was diluted in transport buffer 1∶1000 from stock solution, and centrifuged at 22000×g for 30 minutes. Single FCS runs of 60 seconds length were performed and average values were calculated.

ATTO647N-labeled Oligonucleotides were diluted 1∶10^5^ from stock concentration (1 nmol/µL) with PBS, and measured as described for ovalbumin.

## Supporting Information

Figure S1 3D-reconstruction of the polytene chromosomes and their distribution inside the nucleus of the salivary gland cells of the C.tentans larvae. Inside the nucleus the entire DNA is located in the polytene chromosomes, leaving the nucleoplasm chromatin free. Dashed line indicates the border of the nucleus. Images were taken with a confocal microscope. Scale bar, 15 µm.
(0.77 MB TIF)

Figure S2 hrp36 labelled BR mRNP imaged upon epi-illumination. ATTO647N-labelled hrp36 proteins were microinjected into the nucleus of a salivary gland cell. After 10 min the hrp36 proteins were incorporated within mRNPs. The yellow circle indicates the position of the mRNP. Exposure time 20 ms; scale bar 1.5 µm.
(0.51 MB TIF)

Discussion S1 (0.02 MB DOC)

Movie S1 On the left panel, freely diffusing 500 kDa dextranes diluted in PBS were imaged with epi-illumination at room temperature. On the right panel the same sample was imaged with LSFM. Both movies were recorded with 98.91 Hz and displayed with 30 Hz. For both movies the same camera and laser power settings were used. Field of view is 19.2 µm×19.2 µm.
(6.56 MB AVI)

Movie S2 Three different species of freely diffusing single molecules in buffer were imaged with 483.09 Hz and displayed with 30 Hz. On the left panel 500 kDa dextran molecules with an diffusion coefficient of Ddextran = 8.2 µm^2^/s are shown, in the center panel 43 kDa ovalbumin molecules with DOva = 88 µm^2^/s and on the right panel 30mer oligonucleotides with a D30mer = 130 µm^2^/s. The movies were filtered with a 3×3 Gauss kernel and contrast enhanced. Field of view for each panel is 19.2 µm×19.2 µm.
(9.84 MB AVI)

Movie S3 Hrp36 was microinjected in the nucleus of a salivary gland cell of a C.tentans larva. After 10 min hrp36 proteins were incorporated into mRNPs and became visible at the employed imaging rate of 49.46 Hz. The movie is displayed with 30 Hz. Field of view is 19.2 µm×19.2 µm.
(3.29 MB AVI)

Movie S4 The 500 kDa dextran was microinjected in the nucleus of a salivary gland cell of a C.tentans larva and immediately imaged at a frame rate was 49.46 Hz. The movie is displayed with 30 Hz; field of view, 19.2 µm×19.2 µm.
(3.29 MB AVI)

Materials and Methods S1 (0.02 MB DOC)
