# Supplementary figures and images for: Light Sheet Microscopy for Single Molecule Tracking in Living Tissue
Source: PLoS One. 2010 Jul 23;5(7):e11639. doi: 10.1371/journal.pone.0011639 (PMC2909143; doi:10.1371/journal.pone.0011639)

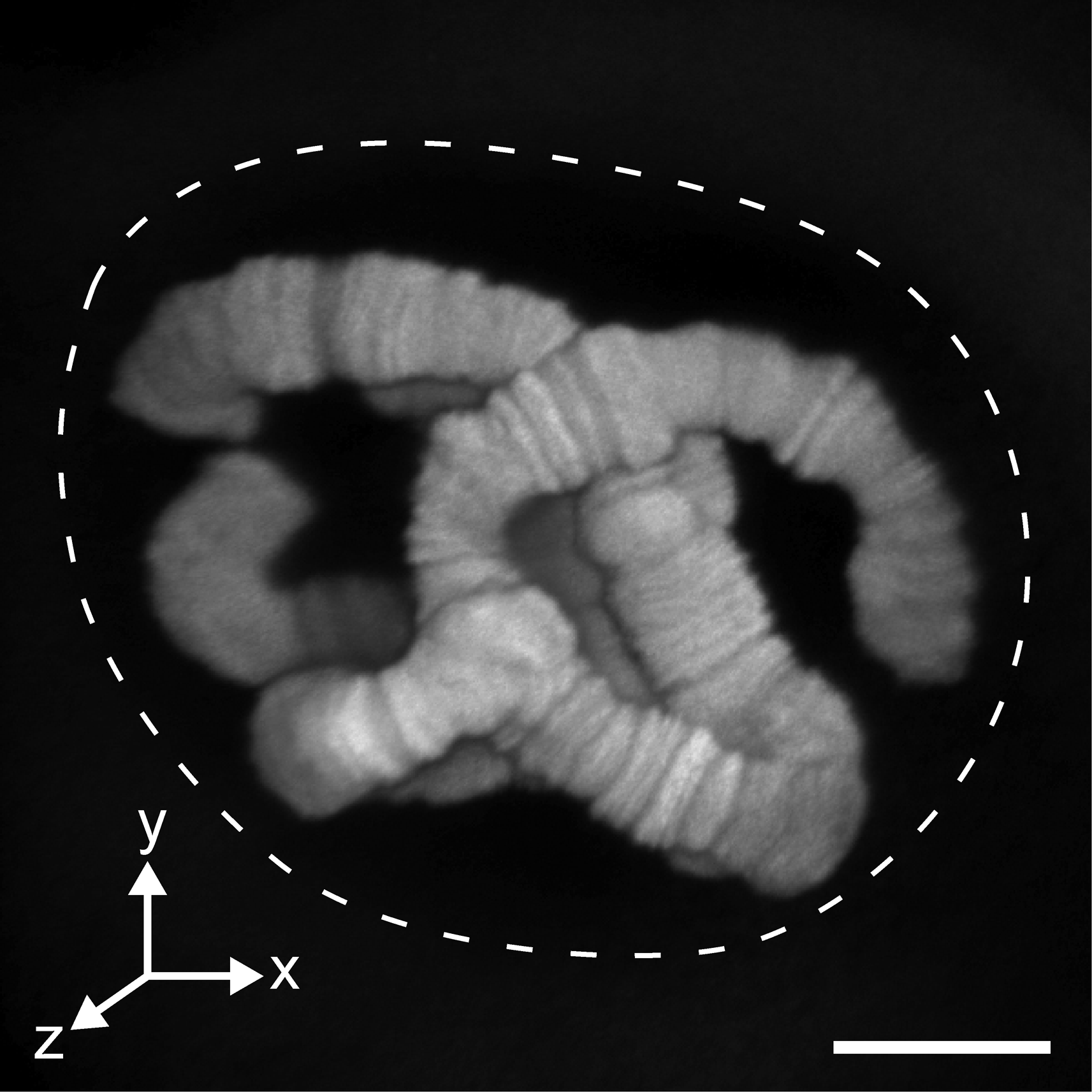

Supplement: Figure S1 — 3D-reconstruction of the polytene chromosomes and their distribution inside the nucleus of the salivary gland cells of the C.tentans larvae. Inside the nucleus the entire DNA is located in the polytene chromosomes, leaving the nucleoplasm chromatin free. Dashed line indicates the border of the nucleus. Images were taken with a confocal microscope. Scale bar, 15 µm. (0.77 MB TIF) [file pone.0011639.s001.tif]

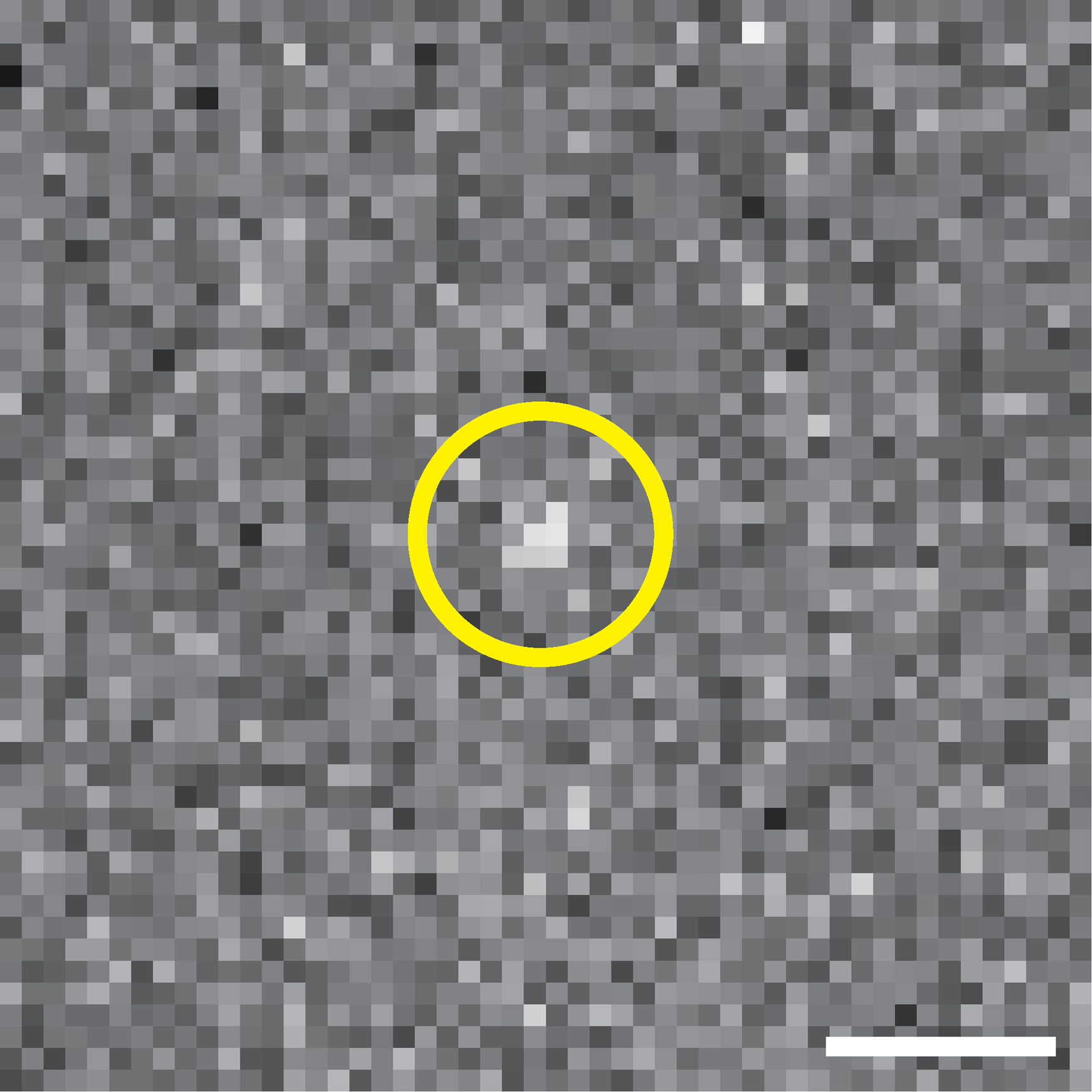

Supplement: Figure S2 — hrp36 labelled BR mRNP imaged upon epi-illumination. ATTO647N-labelled hrp36 proteins were microinjected into the nucleus of a salivary gland cell. After 10 min the hrp36 proteins were incorporated within mRNPs. The yellow circle indicates the position of the mRNP. Exposure time 20 ms; scale bar 1.5 µm. (0.51 MB TIF) [file pone.0011639.s002.tif]
