# Supplementary material for: Light Sheet Microscopy for Single Molecule Tracking in Living Tissue
Source: PLoS One. 2010 Jul 23;5(7):e11639. doi: 10.1371/journal.pone.0011639 (PMC2909143; doi:10.1371/journal.pone.0011639)
Supplement: Discussion S1 — (0.02 MB DOC) [file pone.0011639.s003.doc]

**Discussion S1** In the case of the oligonucleotides distinct deviations from the linear behaviour were observed for times greater than 6 ms (**Fig. 3**). This behaviour was due to the limited size of the observation field. The camera field of view was only 19.2 x 19.2 µm in size, and therefore longer single molecule trajectories were only observable in cases when the respective molecules were confined in this area (1).

1. Grunwald D, Hoekstra A, Dange T, Buschmann V, & Kubitscheck U (2006) Direct observation of single protein molecules in aqueous solution. *Chemphyschem* 7(4):812-815.
