# Supplementary material for: Light Sheet Microscopy for Single Molecule Tracking in Living Tissue
Source: PLoS One. 2010 Jul 23;5(7):e11639. doi: 10.1371/journal.pone.0011639 (PMC2909143; doi:10.1371/journal.pone.0011639)
Supplement: Materials and Methods S1 — (0.02 MB DOC) [file pone.0011639.s008.doc]

**Material & Methods S1** For staining of the polytene chromosomes with propidium iodide the larvae the fixation of the glands was carried out in -20°C cold methanol for 10 minutes, followed by permeabilization with TritonX100 (Carl Roth, Karlsruhe, Germany) for 10 minutes at room temperature. After rinsing with PBS three times, the sample was incubated with propidium iodide (1µg/mL) (Sigma-Aldrich, Germany) for 30 minutes at room temperature. Again the sample was rinsed three times with PBS for 10, 10 and 60 minutes at room temperature. Finally the stained glands were sealed with Vectashield (Vector Laboratories, Burlingame, USA) and an LSM 510 Meta and a NA 1.4 oil immersion objective (Zeiss, Göttingen, Germany) was used for imaging. 3D reconstruction was done with the freely available BioimageXD (www.bioimagexd.net).
